# Value of PCR, Serology, and Blood Smears for Human Granulocytic Anaplasmosis Diagnosis, France

**DOI:** 10.3201/eid2505.171751

**Published:** 2019-05

**Authors:** Yves Hansmann, Benoit Jaulhac, Pierre Kieffer, Martin Martinot, Elisabeth Wurtz, Régis Dukic, Geneviève Boess, André Michel, Christophe Strady, Jean François Sagez, Nicolas Lefebvre, Emilie Talagrand-Reboul, Xavier Argemi, Sylvie De Martino

**Affiliations:** Université de Strasbourg, Strasbourg, France (Y. Hansmann, B. Jaulhac, E. Talagrand-Reboul, S. De Martino);; Hôpitaux Universitaires de Strasbourg, Strasbourg (Y. Hansmann, N. Lefebvre, X. Argemi);; Centre National de Référence des *Borrelia*, Strasbourg (B. Jaulhac, S. De Martino);; Centre Hospitalier Emile Muller, Mulhouse, France (P. Kieffer);; Centre Hospitalier Pasteur, Colmar, France (M. Martinot);; Centre Hospitalier Saverne, Saverne, France (E. Wurtz);; Centre Hospitalier de Haguenau, Haguenau, France (R. Dukic);; Centre Hospitalier de Guebwiller, Guebwiller, France (G. Boess);; Centre Hospitalier de Wissembourg, Wissembourg, France (A. Michel);; Polyclinique Saint André, Reims, France (C. Strady);; Centre Hospitalier Sélestat, Sélestat, France (J.F. Sagez)

**Keywords:** *Anaplasma phagocytophilum*, bacteria, anaplasmosis, tickborne diseases, PCR, serology, blood smears, diagnostics, human granulocytic anaplasmosis, HGA, prospective study, France, vector-borne infections, diagnosis

## Abstract

We prospectively examined the effectiveness of diagnostic tests for anaplasmosis using patients with suspected diagnoses in France. PCR (sensitivity 0.74, specificity 1) was the best-suited test. Serology had a lower specificity but higher sensitivity when testing acute and convalescent samples. PCR and serology should be used in combination for anaplasmosis diagnosis.

Human granulocytic anaplasmosis (HGA) is a tickborne intracellular bacterial infection caused by *Anaplasma phagocytophilum*. The disease is present in North America, Europe, and northern Asia, areas with *Ixodes ricinus* ticks, the primary vector for transmission to humans (*1*,*2*). Clinical manifestations of disease include acute fever, headache, and myalgia occurring 2–3 weeks after tick bite. Diagnosis requires the isolation of *A. phagocytophilum* in blood culture, the presence of morulae in polymorphonuclear cells after May Grünwald-Giemsa staining of peripheral blood smears, positive serologic results (seroconversion or high titer of specific antibodies), or a positive *A. phagocytophilum* PCR result. The May Grünwald-Giemsa stain test has a low sensitivity (*3*); PCR and serology are more widely available, but their diagnostic value is not well established. The aim of our study was to compare the diagnostic values of the available microbiological tests in a prospectively selected series of patients with clinical signs and symptoms consistent with an HGA diagnosis.

## The Study

In this prospective, multicenter study, we enrolled symptomatic patients living in Alsace, a region of northeastern France where tickborne diseases are highly endemic. Patients gave written, informed consent to participate in our study, which was approved by the ethics committee of the University Hospital of Strasbourg (Strasbourg, France).

We included patients if they had 1 of the following combinations of signs and symptoms occurring no more than 4 weeks after a tick bite: 1) fever or other symptom presumed related to a tick bite, 2) fever plus thrombocytopenia with or without leukopenia or elevated liver enzyme levels, 3) thrombocytopenia with or without leukopenia, or 4) elevated liver enzyme levels without fever. The first visit included clinical and epidemiologic evaluations and the collection of blood samples for *A. phagocytophilum* serology, May Grünwald-Giemsa staining, and *A. phagocytophilum*–specific PCR. We did not culture for *A. phagocytophilum*. An etiologic investigation was also conducted to obtain a differential diagnosis. After >4 weeks, a second visit was scheduled to obtain a clinical evaluation, *A. phagocytophilum* serology, and (if necessary) a complete differential diagnosis.

We stratified patients into 3 groups on the basis of their diagnosis. One group included controls, who were patients with a clinical and microbiologically confirmed nonanaplasmosis diagnosis. The second group included anaplasmosis patients defined by >1 of the following criteria: intraleukocyte morulae on blood smears, a positive PCR result for *Anaplasma*, a 4-fold increased antibody titer for *A. phagocytophilum* in the follow-up sample or a seroconversion (i.e., change in antibody titer from negative in first sample to >1:64 in second sample), or a high antibody titer for *Anaplasma* (>1:256) by indirect immunofluorescence antibody assay. The third group were patients without any diagnosis.

We performed DNA extraction, PCR, and serologic testing blinded to sample identification as previously described (*4*). The PCR targeted the *A. phagocytophilum msp2/p44* gene. We performed serologic testing using the *Anaplasma phagocytophilum* IFA IgG assay (Focus Diagnostics, http://www.focusdx.com) (*4*). Trained staff examined May Grünwald-Giemsa–stained smear preparations of whole blood samples for intracellular morulae. We collected data by using EpiData version 3.1.2701.2008 (http://epidata.dk) and extracted data to Excel spreadsheets (Microsoft, https://www.microsoft.com) for analysis. After patient stratification, we estimated the sensitivity and specificity of the different diagnostic tests.

During May 2010–July 2012, we enrolled 155 patients into the study, 25 of whom did not complete the second visit. None of these 25 patients had a positive PCR result or an antibody titer >1:256 at the first visit. The remaining 130 patients completed both study visits and were thus included in the study evaluation. Of these 130 patients, 19 had confirmed anaplasmosis diagnoses and 36 were controls with confirmed nonanaplasmosis diagnoses (infections with *Borrelia burgdorferi*, Epstein-Barr virus, cytomegalovirus, HIV, tick-borne encephalitis virus, *Leptospira* spp., *Babesia* spp., parvovirus B19, hantavirus, *Francisella tularensis*, *Plasmodium* spp., and *Aeromonas* spp.). Of the patients with HGA, 84.2% (16/19) met the serologic criteria and 73.7% (14/19) met the PCR criteria (Table; Figure). Fever, the most frequent symptom (89%), was associated with joint and muscle pain. Cytopenia of platelets, neutrophils, or both (74%) and elevated liver enzyme levels (63%) were frequently present.

**Table:** *Anaplasma phagocytophilum* diagnostic test results of patients with nonanaplasmosis and human granulocytic anaplasmosis diagnoses, France, May 2010–July 2012

Test result	Control group, no./total	*Anaplasma* group, no./total
Positive blood smear	0/36	4/19
Positive by serology	2/36	16/19
Seroconversion* or 4-fold rise in antibody titer	1/36†	6/19‡§
Antibody titer >1:256 at first visit	1/36¶	11/19§
Positive PCR	0/36	14/19

*Seroconversion is defined as a change in antibody titer from negative in the first sample obtained during acute illness to >1/64 in the second sample acquired >4 weeks later. †One patient had a seroconversion with a microbiologically confirmed diagnosis of parvovirus B19 infection. ‡Only 1 patient had a 4-fold increase in antibody titer, but the titer at the first study visit was already high enough to establish the diagnosis (increase from 1:512 to 1:2,048).  §One patient had a seroconversion with an *A. phagocytophilum* antibody titer >1:256 at the second visit (patient counted once in both serology categories). All other patients with seroconversion had an antibody titer <1:256. ¶One patient with microbiologically confirmed leptospirosis had an *A. phagocytophilum* antibody titer of 1:256 at the first visit that decreased to 1:64 at the second visit.

**Figure:**
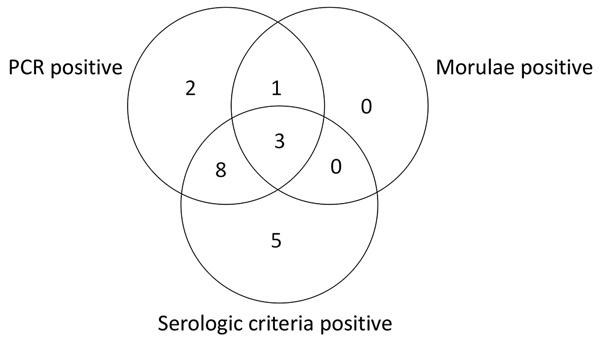
Distribution of positive diagnostic test results for patients with confirmed human granulocytic anaplasmosis, France, May 2010–July 2012.

Calculations of the diagnostic value of each test method showed that PCR had a sensitivity of 0.74 and a specificity of 1 and blood smear staining had a sensitivity of 0.21 and a specificity of 1. Seroconversion or a 4-fold increase of antibody titer had a sensitivity of 0.32 and specificity of 0.97, an antibody titer >1:256 had a sensitivity of 0.58 and specificity of 0.97, and overall serology had a sensitivity of 0.84 and specificity of 0.97.

The interval between the first and second serologic tests for most patients in the anaplasmosis group was 4–8 weeks (mean 49.8 days). Five patients had the second test >8 weeks after the first. Of these patients, 2 seroconverted, 1 experienced a substantial decrease in antibody titer, 1 experienced a substantial increase at week 12, and 1 had a stable antibody titer.

Our study confirms PCR as the gold standard for diagnosis of HGA; this test enabled rapid diagnosis during the acute stage of infection with good sensitivity and excellent specificity. However, the absence of a gold standard diagnostic test to compare our results with is a limitation to our study. *A. phagocytophilum* culture is the reference test for HGA diagnosis (*5*,*6*) but is not well suited for routine use because culturing is time-consuming and not widely performed. The diagnosis of anaplasmosis often involves assessing for the presence of morulae, but this test has low sensitivity (*3*). In our study, this test was of limited value for HGA diagnosis because whenever morulae were detected on blood smears >1 of the other diagnostic tests were positive. However, May Grünwald-Giemsa staining is the quickest test to do, and when performed by trained staff, positive results are helpful for physicians.

In clinical practice, diagnosis of HGA often relies on serology (*7*–*9*), but 2 limitations are associated with this method: a risk for false-negative results during the acute stage of infection because *A. phagocytophilum* antibodies are detected on average 11.5 days after symptom onset and a risk for false-positive results because *Anaplasma* antibodies are detectable in 86.4% of patients for 6–10 months and in 40% of patients up to 2 years after the initial infection (*10*). Positive serologic criteria are seroconversion, a 4-fold increase in antibody titer, or a stable and high antibody titer (*11*,*12*). In our study, we observed that each of these criteria can lead to misdiagnosis at the beginning of infection, as previously reported (*13*).

PCR is considered the most effective diagnostic method during early stage *A. phagocytophilum* infection (*14*,*15*). Our results confirm this belief, despite our limitation of a small study population. However, if PCR is use alone, HGA might be underdiagnosed.

## Conclusions

The presentation of fever in a patient with a history of tick bite does not qualify for an anaplasmosis diagnosis; microbiological tests need to be performed. For anaplasmosis, PCR testing appears to be the most effective diagnostic tool. However, the sensitivity of PCR is <100%, and combining PCR with serologic testing at the first visit appears to be the best strategy for early diagnosis of acute anaplasmosis. In cases of high suspicion for HGA in patients without any diagnosis at the first visit, a second serologic test >4 weeks later can be helpful. A multiplex approach could also be used in such cases to look for differential diagnoses.
